# Supplementary material for: Dynamic changes in mitochondria support phenotypic flexibility of microglia
Source: Nat Commun. 2025 Dec 12;16:11103. doi: 10.1038/s41467-025-66709-5 (PMC12700904; doi:10.1038/s41467-025-66709-5)
Supplement: Supplementary file 1 — Supplementary Information [file 41467_2025_66709_MOESM1_ESM.pdf]

Supplementary Figure 1 - Related to Figure 1

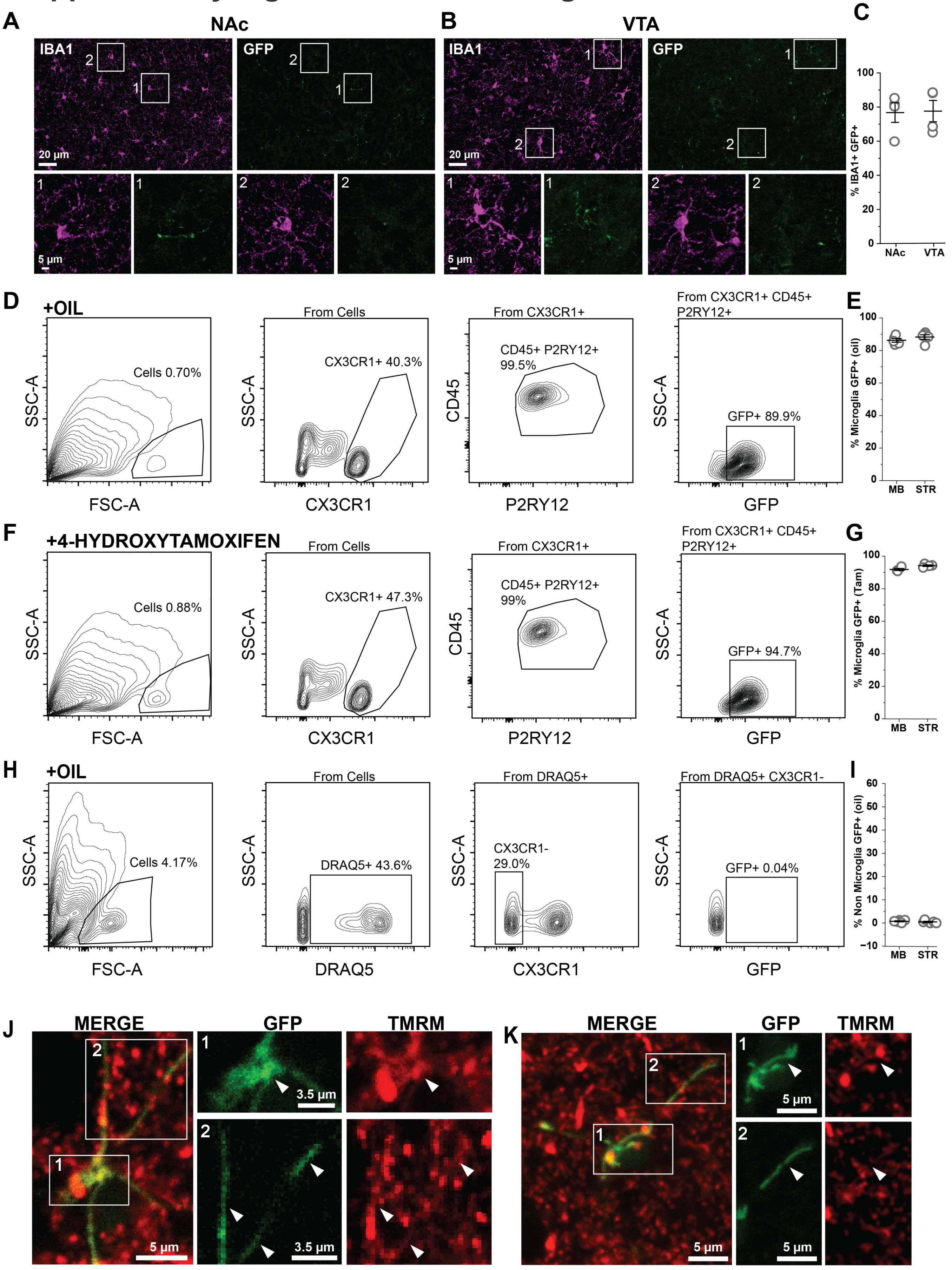

FIGURE S1: *MG-MitoGFP* mice accurately label microglial mitochondria (*related to Figure 1*). (A,B) 20x confocal images of native GFP signal and Iba1 (*magenta*) staining in *CX3CR1-CreER; flox-stop mitoGFP* (*MG-MitoGFP*) mice. *Boxes* highlight examples of IBA1+ GFP+ positive cells as well as IBA1+GFP- cells from both the NAc and VTA. **(C)** Percentage of Iba1+ NAc and VTA microglia exhibiting GFP expression. N = 4 mice for NAc and VTA, 2-3mo of age. Data was plotted as mean +/- SEM. **(D)** Sequential gating strategy for FACS-based analysis of microglia from oil-injected *MG-MitoGFP* mice. **(E)** Percentage of midbrain (MB) and striatum (STR) microglia (CX3CR1+P2RY12+CD45+) that are also GFP+. N = 5 mice for MB and STR, 3-4mo of age. Data was plotted as mean +/- SEM. **(F)** Sequential gating strategy for FACS-based analysis of microglia from 4-hydroxytamoxifen-injected *MG-MitoGFP* mice. **(G)** Percentage of MB and STR microglia (CX3CR1+P2RY12+CD45+) that are also GFP+ in 4-hydroxytamoxifen treated mice. N = 4 mice for MB and STR, 3-4mo of age. Data was plotted as mean +/- SEM. **(H)** Sequential FACS gating strategy used to identify non-microglial cells (DRAQ5+, CX3CR1-) and examine GFP expression. **(I)** Percentage of non-microglial cells (CX3CR1- Draq5+) exhibiting GFP expression in MB and STR. N = 4 mice for MB and STR, 3-4mo of age. Data was plotted as mean +/- SEM. **(J-K)** Live imaging of acute brain sections from *MG-MitoGFP* mice incubated with mitochondrial membrane potential indicator dye TMRM. Source data for all graphs are provided as a Source Data file.

Supplementary Figure 2 - Related to Figure 1

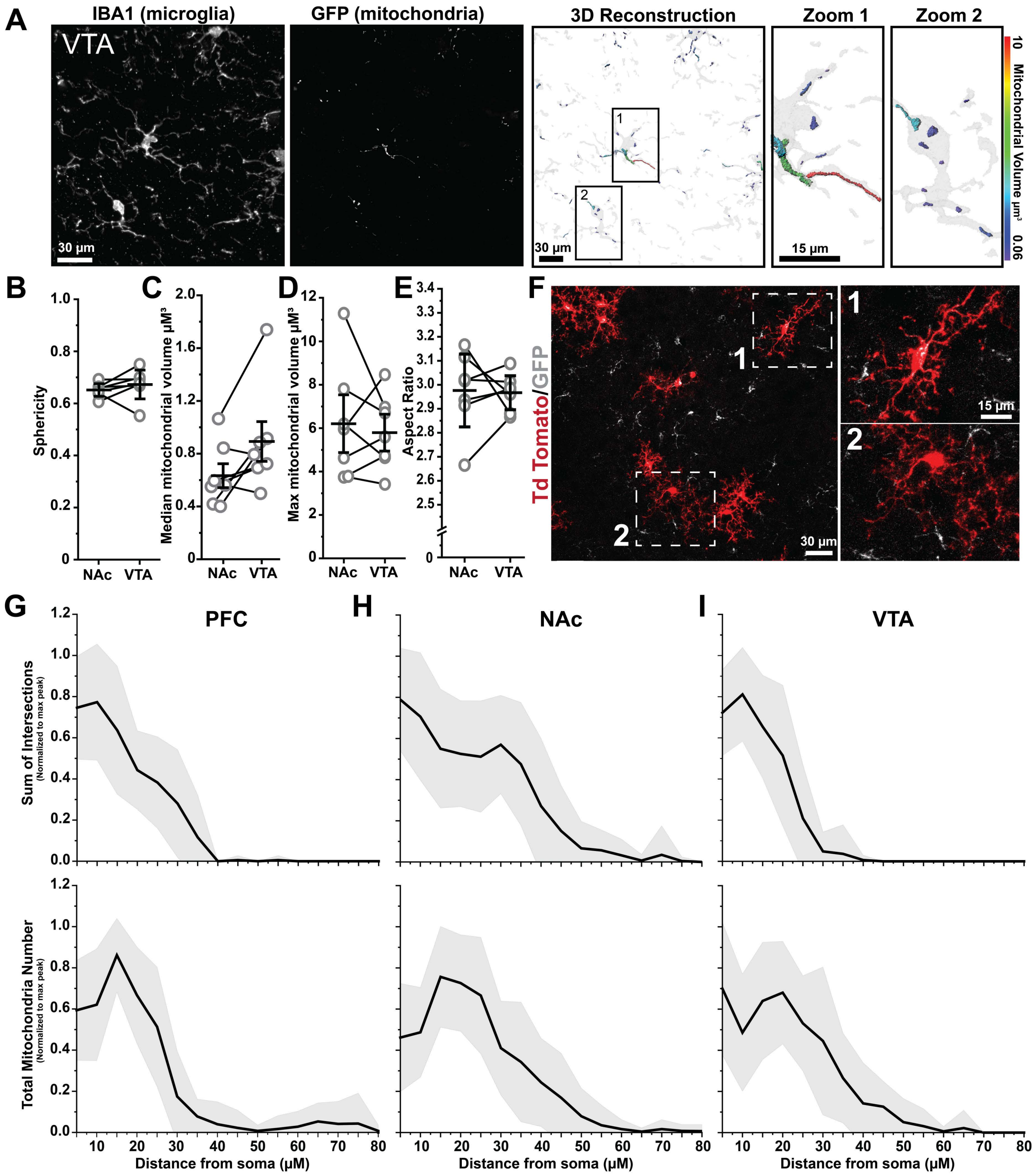

J

Morphological Parameters	Region	Total # of Mitochondria	Total Volume of Mitochondria
Total Process Length	PFC	R=0.49 P=0.05 n.s.	R=-0.02 P=0.92
	Nac	R=0.14 P=0.48	R=0.49 P=0.03 *
	VTA	R=0.48 P=0.07	R=0.40 P=0.11
Sum of branch pts.	PFC	R=0.40 P=0.17	R=-0.17 P=0.59
	Nac	R=0.13 P=0.42	R=0.45 P=0.08
	VTA	R=0.56 P=0.02*	R=0.49 P=0.08
Max # of intersections	PFC	R=0.54 P=0.02*	R=-0.16 P=0.99
	Nac	R=0.45 P=0.08	R=-0.34 P=0.13
	VTA	R=0.53 P=0.05 n.s.	R=0.48 P=0.10

FIGURE S2: Mitochondrial abundance and distribution align with morphological features of microglia (*related to Figure 1*). (A) Representative high-magnification images of VTA microglia (*Iba1*) and mitochondria (*native GFP*); 3D volumetric reconstructions of microglia (*gray*) and mitochondria (*colored according to volume*) from *MG-MitoGFP* mice. **(B-E)** Quantification of field of view (FOV) mitochondrial morphological parameters across brain regions. Data points from the same mouse are identified by connecting lines (N = 7 mice, 2mo of age). Sphericity: P = 0.346, Two-tailed paired t-test. Median mitochondria volume: P = 0.056, Two-tailed paired t-test. Max mitochondria volume: P = 0.640, Two-tailed paired t-test. Aspect ratio (longest axis divided by shortest axis): P = 0.894, Two-tailed paired t-test. *P<0.05 Data was plotted as mean +/- SEM. **(F)** Representative 20x image of the sparse recombination observed in *MG-MitoGFP;Ai14* triple transgenic mice that have not been treated with 4-hydroxytamoxifen. **(G-I)** Sholl analysis of 3D-reconstructed microglia and distribution of their mitochondria relative to cell somas (PFC N = 16, NAc N = 16 and VTA N = 15 cells). Data was plotted as mean (black line) +/- SEM (gray shading). **(J)** Table of robust regression analyses relating cell morphological features of individual microglia to number and volume of their mitochondria. Mice used for these experiments were not treated with 4-hydroxytamoxifen. Source data used to prepare all graphs are provided as a Source Data file.

Supplementary Figure 3 - Related to Figure 2

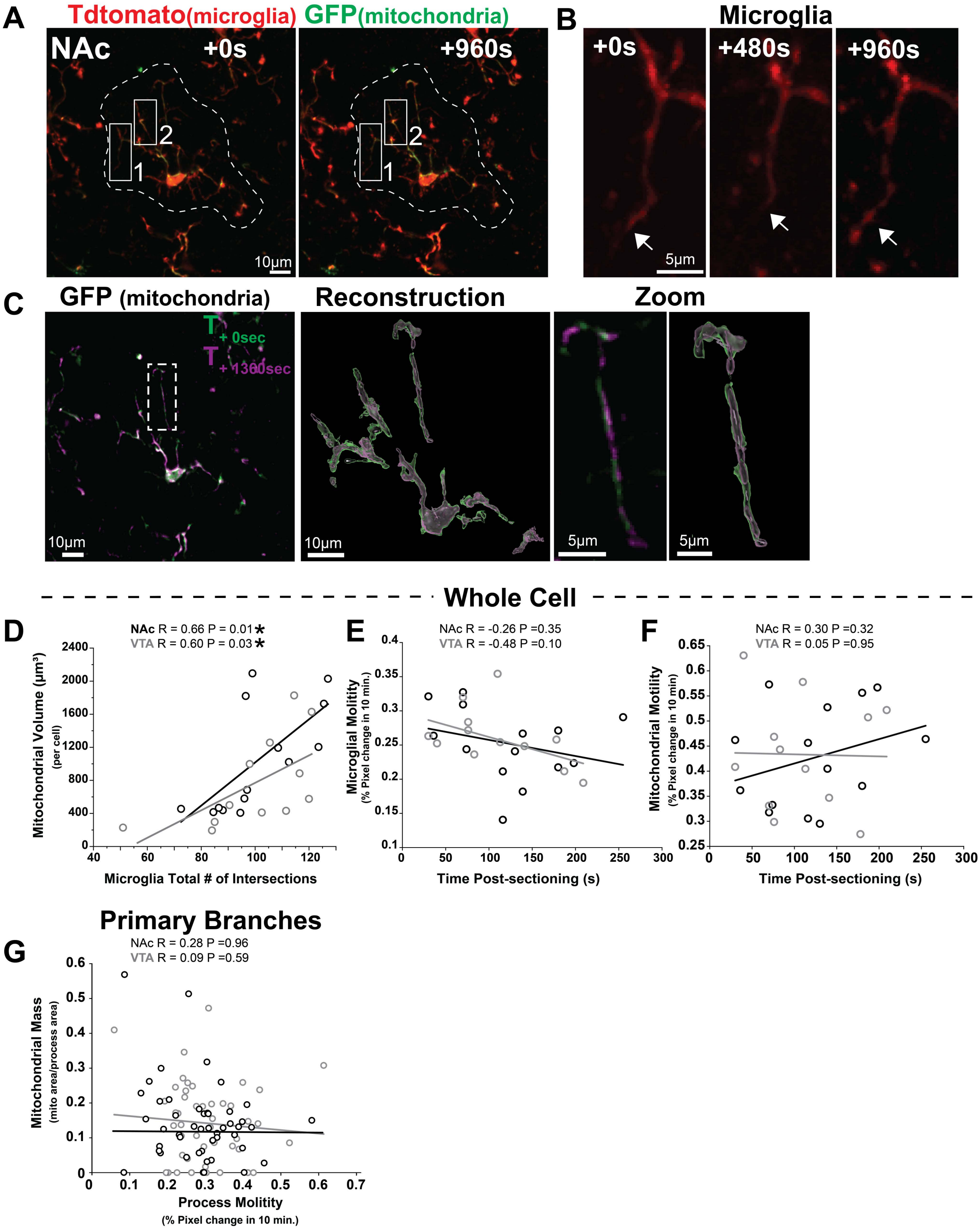

FIGURE S3: Microglial motility is not correlated with tissue sectioning or mitochondrial motility (*related to Figure 2*). (A) Representative images taken during multiphoton live imaging of NAc microglia (*TdTomato*) in acute brain sections from 1.5 -2mo old *MG-mitoGFP;Ai14* mice. **(B)** Example of microglial cell process retraction and re-extension highlighted by *white arrows*. **(C)** Raw fluorescence from multiphoton imaging and corresponding 3D reconstruction of microglial mitochondria, colored by time. *Dashed white box* shown at higher magnification *at right*. **(D)** Robust regression with false discovery rate correction analysis relating total mitochondrial volume per cell and microglial morphological complexity (total Sholl intersections across all radii). N = 14 NAc cells, R = 0.66 P = 0.01 and N = 12 VTA cells, R = 0.60 P = 0.03. **(E)** Robust regression with false discovery rate correction analysis relating microglial motility (% pixel change over 10min) to time elapsed since preparation of acute brain sections. N = 14 NAc cells, R = -0.26 P = 0.35 and N = 12 VTA cells, R = -0.48 P = 0.10. **(F)** Robust regression with false discovery rate correction analysis relating mitochondrial motility (% pixel change over 10min) to time elapsed since preparation of acute brain sections. N = 14 NAc cells, R = 0.30 P = 0.32 and N = 12 VTA cells, R = 0.05 P = 0.95. **(G)** Robust regression with false discovery rate correction analysis relating mitochondrial mass within one primary microglial branch to motility of that primary branch for NAc and VTA microglia. N = 55 NAc microglia primary processes, R = 0.28 P = 0.96 and N=46 VTA microglia primary processes, R = 0.09 P = 0.59. Mice used for these experiments were not treated with 4-hydroxytamoxifen. *P<0.05. Source data used to prepare all graphs are provided as a Source Data file.

Supplementary Figure 4 - Related to Figure 4

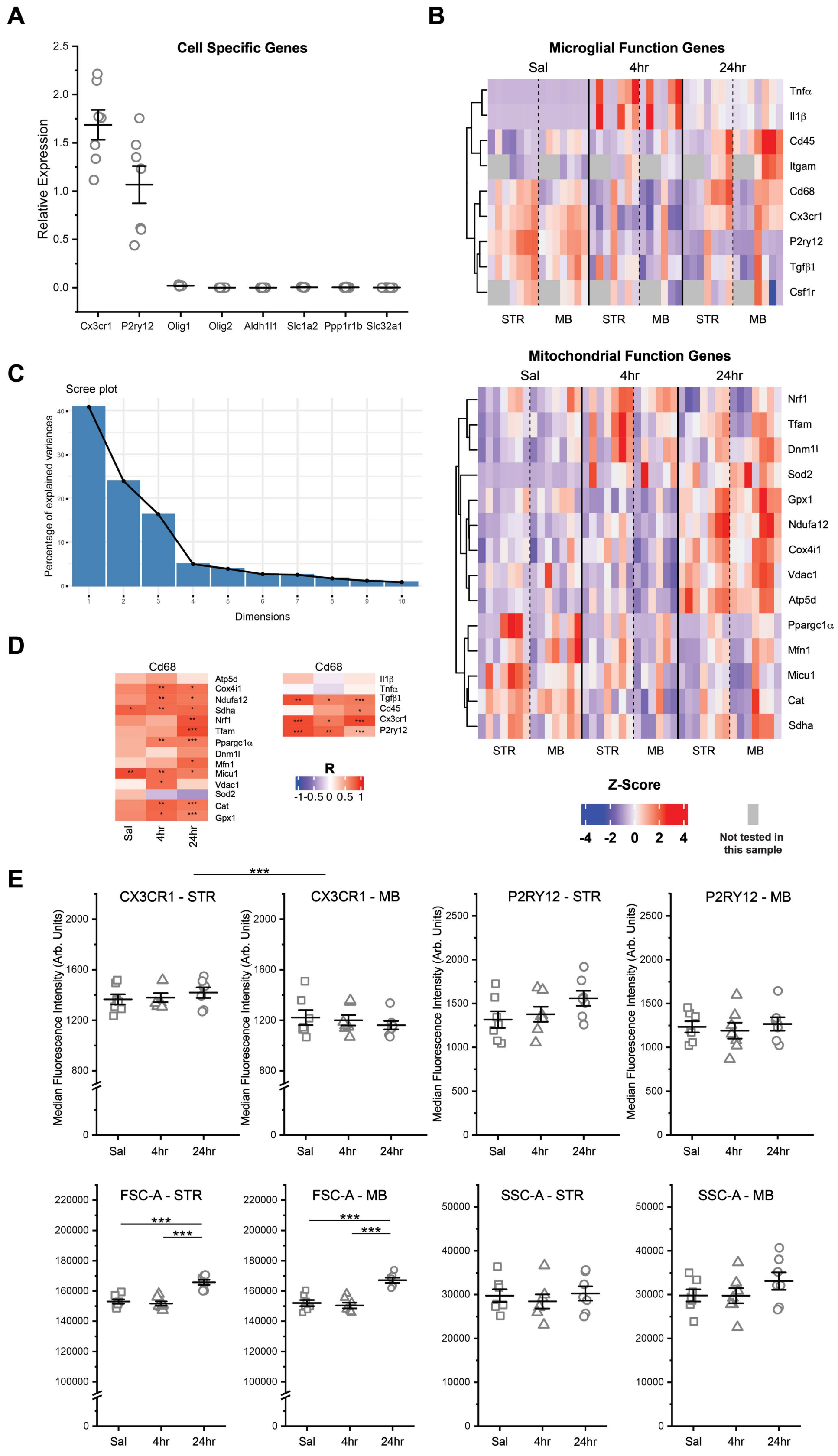

FIGURE S4: Microglial and mitochondrial responses to inflammatory challenge (LPS, *related to Figure 4*). (A) Relative expression levels ($2^{-\Delta Ct}$) of cell specific genes for microglia (*Cx3cr1* and *P2ry12*), oligodendrocyte lineage cells (*Olig1*, *Olig2*), astrocytes (*Aldh1l1*, and *Slc1a2*) and neurons (*Ppp1r1b* and *Slc32a1*) from a subset of saline and LPS treated samples (N = 7 mice), confirming purity of collected microglia. Data was plotted as mean +/- SEM. **(B)** Heatmaps showing relative expression levels ($2^{-\Delta Ct}$) of key microglial function and mitochondrial function genes for individual samples (Saline STR N = 7 mice, Saline MB N = 7 mice, LPS 4hr STR N = 7 mice, LPS 4hr MB N = 6 mice, LPS 24hr STR N = 7 mice, LPS 24hr MB N = 7 mice); *corresponding heat map with group averages shown in Fig. 4B*. **(C)** Scree plot demonstrating the percentage of dataset variance explained by each principal component identified via Principal Component Analysis of all samples. PCA (shown in *Fig. 4C*) was carried out using expression values from mitochondrial function genes only. **(D)** Heat maps depicting the degree of correlation between *Cd68* expression and expression levels of microglial and mitochondrial function genes as determined by robust regression analysis with false discovery rate correction. See *Table S4* for R^2- and P-values. * P < 0.05, ** P < 0.01, *** P < 0.001. **(E)** Median Fluorescence Intensity of CX3CR1, P2RY12, FSC-A, SSC-A signals in STR and MB microglia from Saline (N = 7, *squares*), LPS 4hr (N = 7, *triangles*), and LPS 24hr (N = 7, *circles*) mice. Two-way ANOVA with Bonferroni correction for post hoc comparisons: CX3CR1, main effect of treatment, $F_{(2,36)} = 0.00442$, P = 0.99559, main effect of region, $F_{(1,36)} = 30.80804$, P < 0.0001; P2RY12, main effect of treatment, $F_{(2,36)} = 1.71502$, P = 0.19514, main effect of region $F_{(1,36)} = 7.52567$, P = 0.00942; FSC-A, main effect of treatment, $F_{(2,36)} = 49.45017$, P < 0.0001, main effect of region, $F_{(1,36)} = 0.03974$, P = 0.84311; SSC-A, main effect of treatment, $F_{(2,36)} = 1.32646$, P = 0.27808, main effect of region, $F_{(1,36)} = 1.07222$, P = 0.30735. *** P < 0.001. Data was plotted as mean +/- SEM. Mice used for these experiments were not treated with 4-hydroxytamoxifen. Source data used to generate all graphs are provided as a Source Data file.

Supplementary Figure 5 - Related to Figure 5

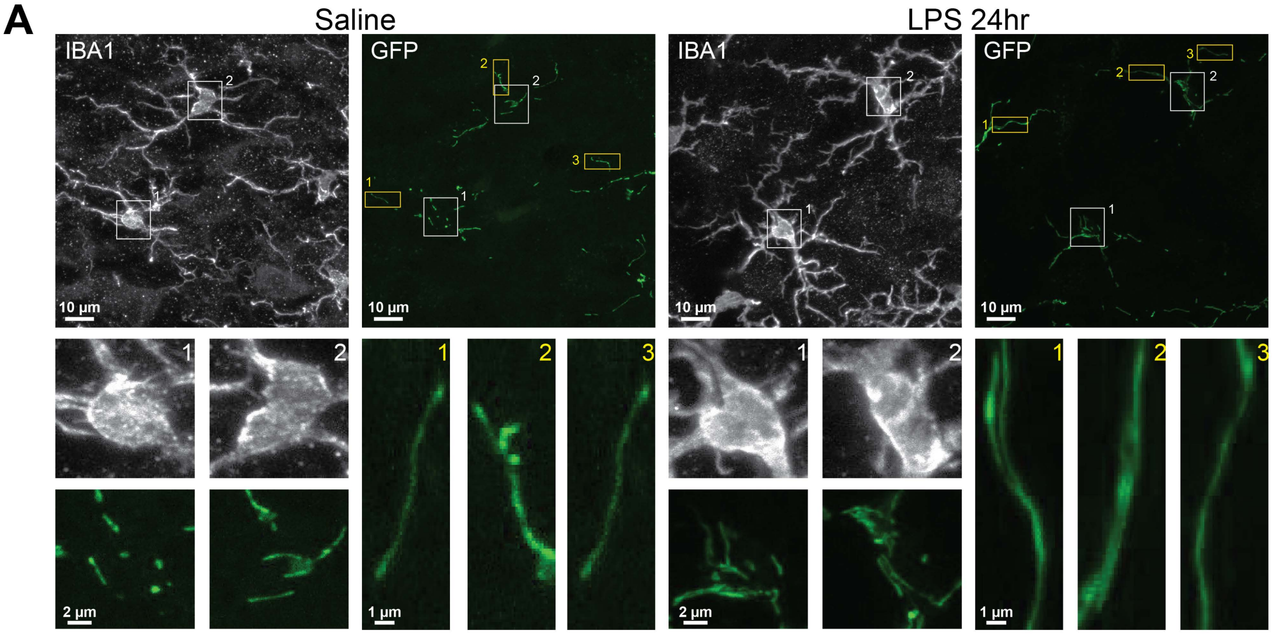

FIGURE S5: VTA microglial mitochondrial response to inflammatory challenge (LPS, *related to Figure 5*). (A) Representative images of VTA microglia (IBA1) and microglial mitochondria (GFP) from 3-4mo *MG-MitoGFP* mice injected with saline (*left*) or LPS 24hr (*right*) prior to euthanasia. *White* and *yellow boxes* highlight examples of somatic mitochondria and cell process mitochondria shown at higher magnification *below*. Mice used for these experiments were not treated with 4-hydroxytamoxifen.

Supplementary Figure 6 - Related to Figure 6

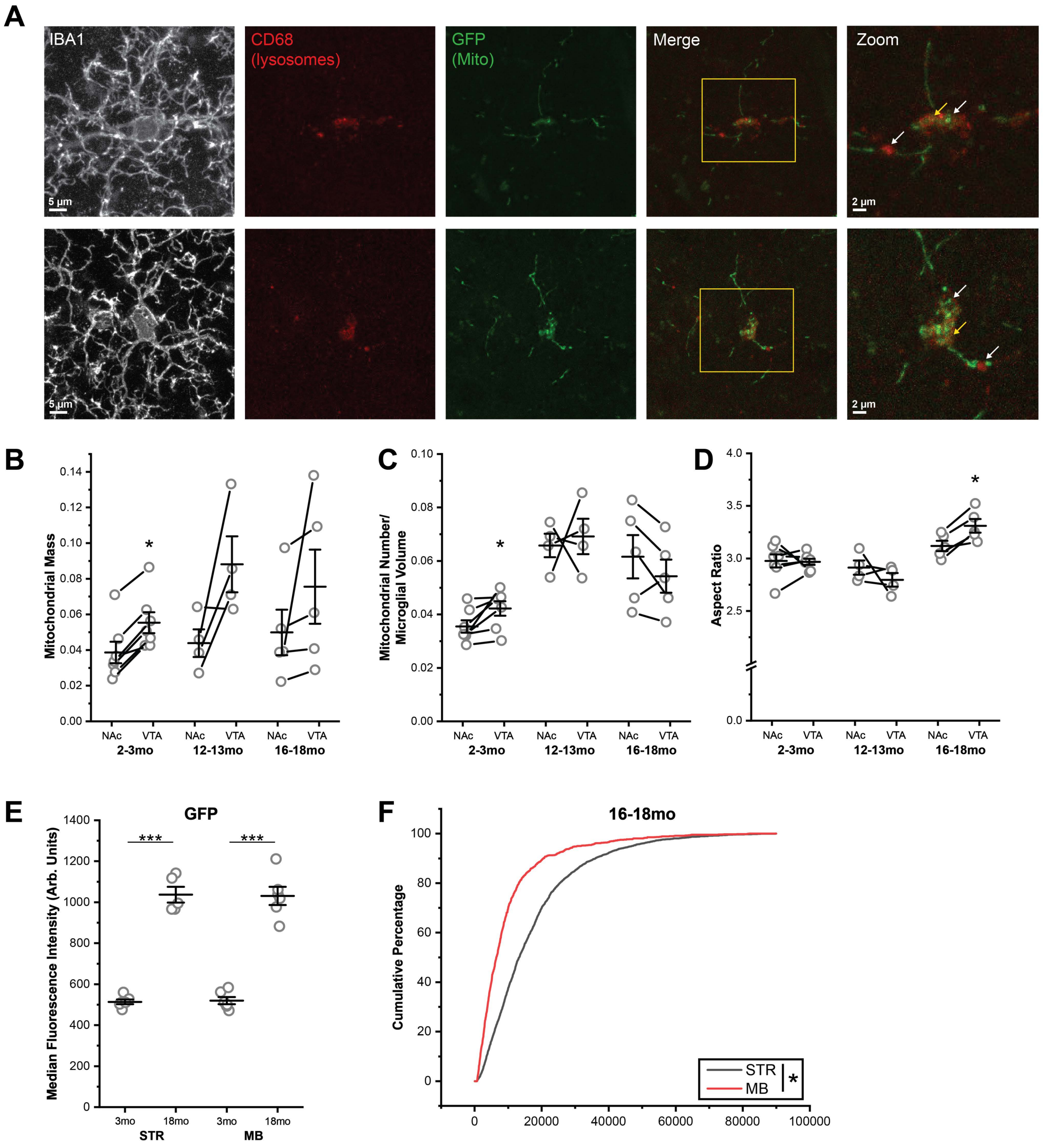

FIGURE S6: Microglial mitochondria undergo remodeling in middle- and late middle aged mice (*related to Figure 6*). (A) Representative confocal images of microglia (IBA1) from 18mo *MG-MitoGFP* mice revealing dim remnants of somatic autofluorescent material following tissue treatment with Trueblack (Biotium). Co-staining with CD68 confirms that brighter mitoGFP signals (*white arrows*) are not colocalized with bright CD68 puncta (*white arrowheads*) and that dimmer somatic GFP autofluorescence that colocalizes with CD68 (*yellow arrows*) is easily distinguished from brighter mitoGFP signals. **(B-D)** Comparison of FOV microglial mitochondrial mass (2-3mo P = 0.001, 12-13mo P = 0.092, 16-18mo P = 0.235, two tailed paired t-test), mitochondrial number (2-3mo P = 0.026, 12-13mo P = 0.778, 16-18mo P = 0.167, two tailed paired t-test), and mitochondrial aspect ratio (2-3mo P = 0.885, 12-13mo P = 0.392, 16-18mo P = 0.009, two tailed paired t-test) across the NAc and VTA of individual 2-3mo (N = 7 mice), 12-13mo (N = 4 mice), and 16-18mo (N = 5 mice) *MG-MitoGFP* mice. * P< 0.05. Data was plotted as mean +/- SEM. **(E)** GFP median fluorescence intensity of STR and MB microglia from 3mo (N = 6 STR, 6 MB) and 18mo (N = 5 STR, 6 MB) *MG-MitoGFP* mice. Two-way ANOVA with Bonferroni correction for post hoc comparisons: main effect of age, $F_{(1,19)}$ = 287.28, P < 0.0001; main effect of region, $F_{(1,19)}$ = 0.0003, P = 0.9953. *** P < 0.001. Data was plotted as mean +/- SEM **(F)** Cumulative probability distributions comparing microglial TMRM intensity of microglia across brain regions (STR *black*, MB *red*) in 16-18mo C57Bl6 wildtype mice. * P < 0.05, Kolmogorov-Smirnov tests. Mice used for these experiments were not treated with 4 hydroxytamoxifen. Source data are provided as a Source Data file.

Supplementary Figure 7 - Related to Figure 7

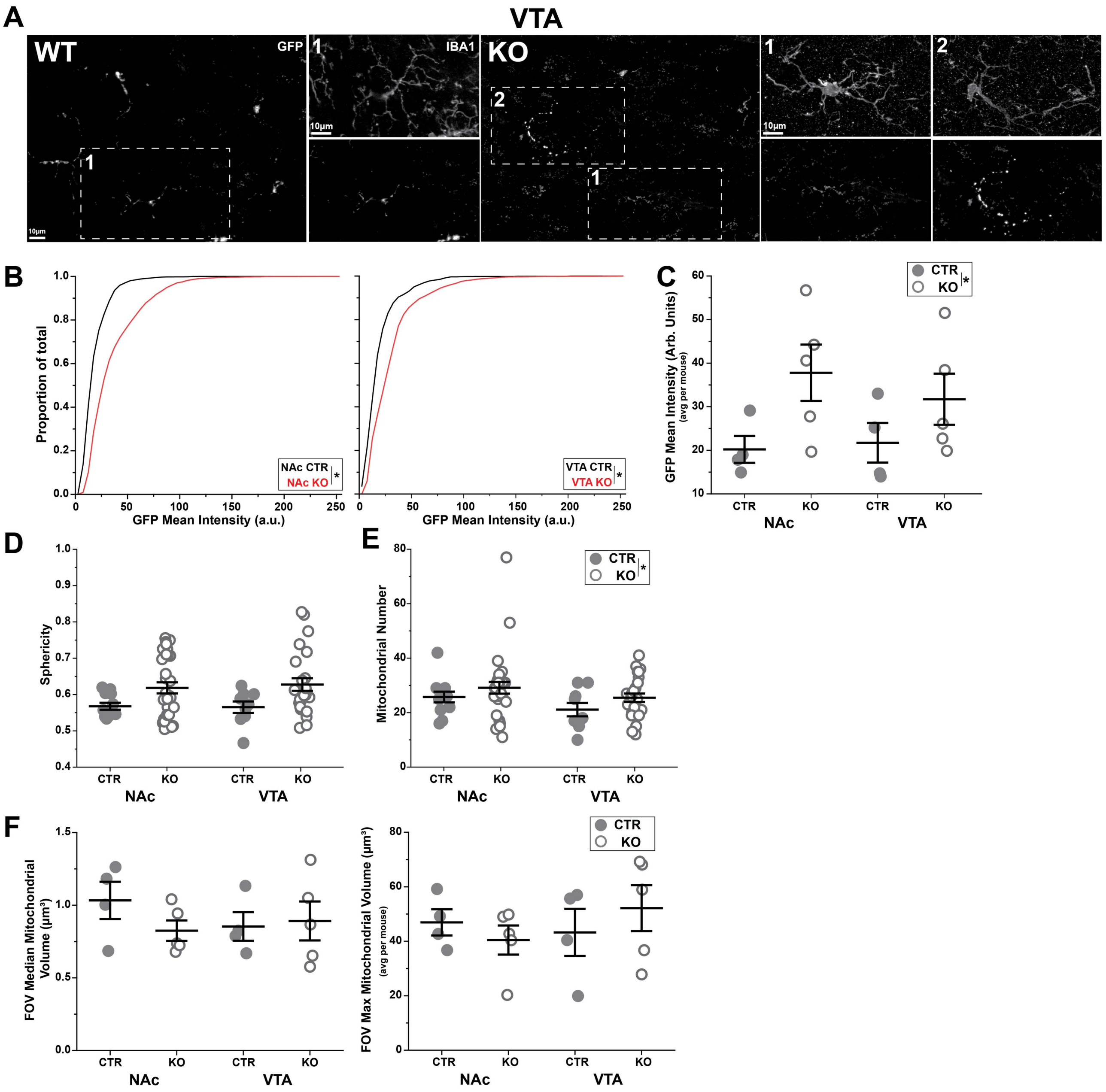

FIGURE S7: Microglial mitochondria display increased GFP intensity in *MG-mitoGFP-TFAMKO* mice (*related to figure* 7). (A) Representative confocal images of microglia (IBA1) and mitochondria (GFP) in the VTA of *MG-mitoGFP* (CTR) and *MG-mitoGFP-TFAMKO* (KO) mice. **(B)** Cumulative distribution of GFP intensity of individual mitochondria across genotypes (CTR = *black*, KO = *red*) in the VTA and NAc. Distribution curves for each animal within a group were averaged to generate the displayed, group average, cumulative distributions. *P < 0.05, Kolmogorov-Smirnov test. **(C)** Mean mitoGFP intensity for all mitochondria analyzed for each mouse. CTR (*filled circles*) = 4 mice and KO (*open circles*) = 5 mice. Two-way ANOVA with Bonferroni correction for post hoc comparisons: main effect of genotype, $F_{(3,14)} = 6.264$, P = 0.025, main effect of brain region, $F_{(3,14)} = 0.171$, P = 0.685. Data was plotted as mean +/- SEM. **(D)** Average sphericity of mitochondria within individual NAc and VTA microglial cells. Two-way ANOVA with Bonferroni correction for post hoc comparisons: main effect of genotype $F_{(3,73)} = 8.119$, P = 0.005, main effect of brain region, $F_{(3,73)} = 0.028$, P = 0.867. Data was plotted as mean +/- SEM. **(E)** Total number of mitochondria within individual NAc and VTA microglial cells. Two-way ANOVA with Bonferroni correction for post hoc comparisons: main effect of genotype $F_{(3,73)} = 2.452$, P = 0.121, main effect of brain region, $F_{(3,73)} = 2.805$, P = 0.098. Data was plotted as mean +/- SEM. **(F)** Median volume and maximum volume of mitochondria within FOV across CTR (*filled circles*) and KO (*open circles*) mice. Two-way ANOVA with Bonferroni correction for post hoc comparisons: medium volume main effect of genotype $F_{(3,14)} = 0.584$, P = 0.457 and maximum volume main effect of genotype $F_{(3,73)} = 0.254$, P = 0.621. Data was plotted as mean +/- SEM. Mice were treated with 4-hydroxytamoxifen and analyzed 2 months later as described in methods. Source data used to generate all graphs are provided as a Source Data file.

Supplementary Figure 8 - Related to Figure 8

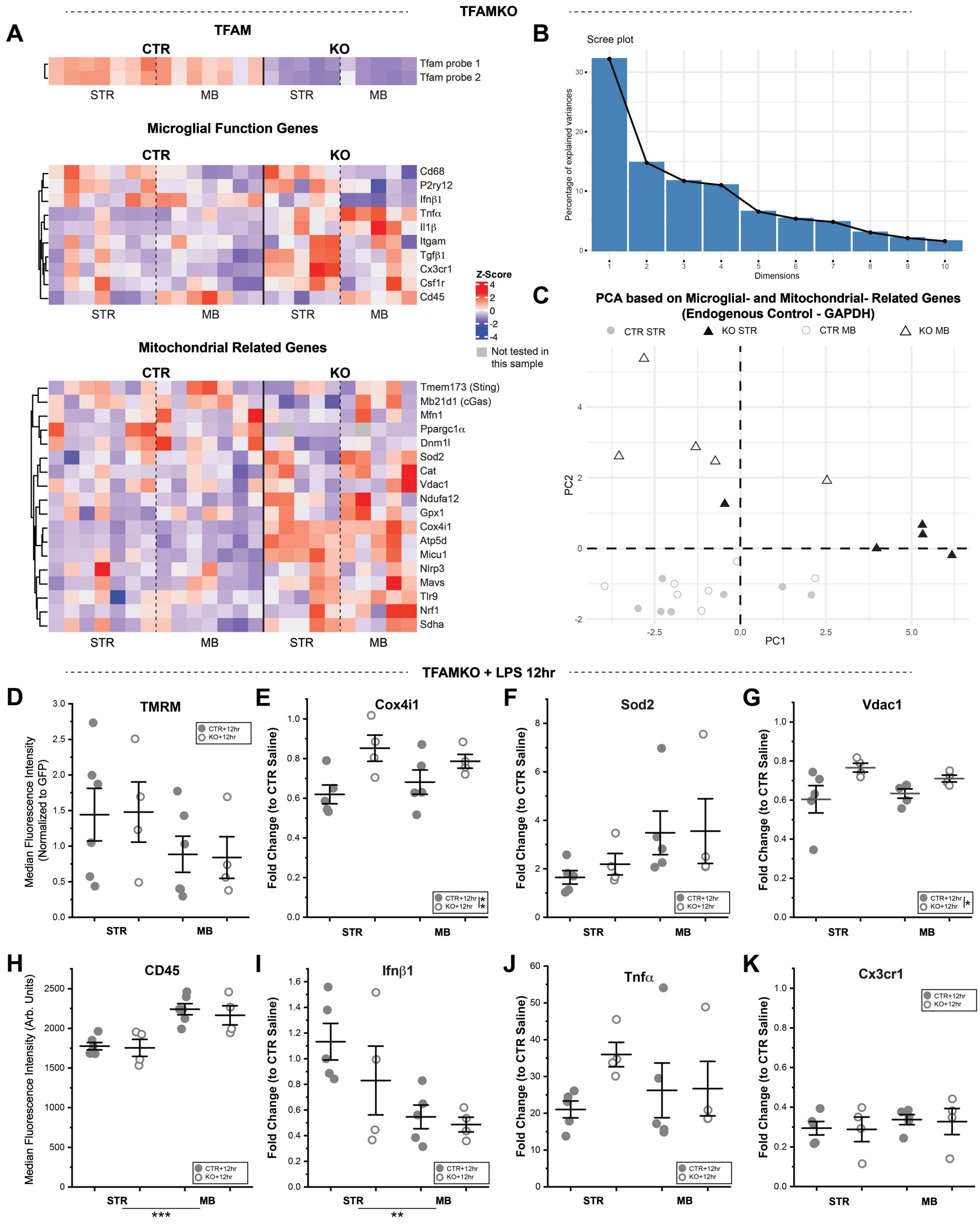

FIGURE S8: TFAM loss alters microglial size and baseline gene expression, but causes only subtle changes in microglial capacity to respond to acute inflammatory challenge (*related to Figure 8*). (A) Heatmaps showing relative expression levels ($2^{-\Delta Ct}$) of *Tfam*, key microglial function genes, and mitochondrial function genes for individual samples (STR and MB from N = 7 CTR mice, STR and MB from N = 5 TFAM KO mice) . **(B)** Scree plot demonstrating the percentage of dataset variance explained by each principal component identified via Principal Component PCA of all samples shown in *Fig. 8C*. **(C)** Principal Component Analysis of microglia from control (STR *filled gray circles,* MB *open gray circles*) and *MG-TFAMKO* (STR *filled black triangles,* MB *open black triangles*) mice based on expression of microglial- and mitochondrial- function genes calculated relative to a second endogenous control gene, *Gapdh*. **(D)** TMRM median fluorescence intensity (normalized to individual cell mitoGFP signal to account for mitochondrial abundance) of microglia from LPS treated CTR (N = 6, *filled circles*) and *MG-TFAMKO* (N = 4, *open circles*) mice. **(E-G)** Fold change ($2^{-\Delta\Delta Ct}$) of expression of mitochondrial genes *Cox4i1*, *Sod2*, and *Vdac1* in LPS treated MG-TFAMKO (N = 4, *open circles*) and LPS treated CTR (N = 5, *filled circles*) mice relative to microglia from saline treated *MG-mitoGFP* mice (*Fig. 4B*). **(H)** CD45 median fluorescence intensity of microglia from LPS treated CTR (N = 6, *filled circles*) and *MG-TFAMKO* (N = 4, *open circles*) mice. **(I-K)** Fold change ($2^{-\Delta\Delta Ct}$) of expression of microglial function genes *Ifnβ1*, *Tnfα*, and *Cx3cr1* in LPS treated MG-TFAMKO (N = 4, *open circles*) and LPS treated CTR (N = 5, *filled circles*) mice relative to microglia from saline treated *MG-mitoGFP* mice (*Fig. 4B*). See *Table S5* and *Table S7* for Two-way ANOVA with Bonferroni correction for post hoc comparisons F^2 and P values. * $P < 0.05$, ** $P < 0.01$, *** $P < 0.001$. Mice were treated with 4-hydroxytamoxifen and analyzed 2 months later as described in methods. Data was plotted as mean ± SEM. Source data used to generate all graphs are provided as a Source Data file.

Supplementary Figure 9

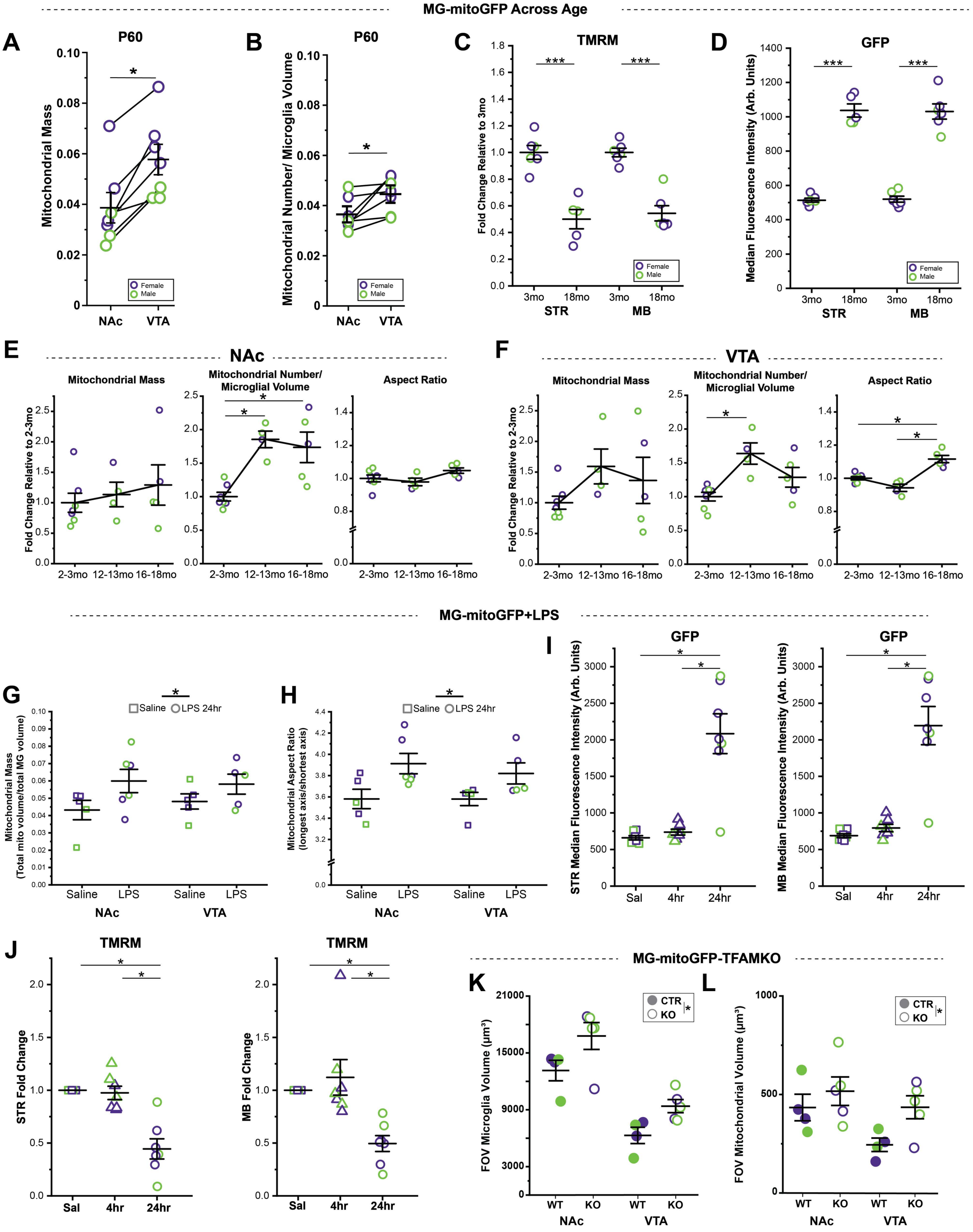

FIGURE S9: Most analyses do not suggest obvious sex-based differences in microglial mitochondrial features (*related to Figures 1, 4, 5, 6, S6, and 7*). Key graphs from the study annotated according to mouse sex with females labeled in *purple* and males in *green* **(A-B)** Mitochondrial mass (mitochondrial volume / microglial volume) and mitochondrial number (relative to cell volume) in NAc (N = 3 male, 4 female mice) and VTA (N = 3 male, 4 female mice). **(C-D)** Median fluorescence intensity of TMRM (normalized to individual cell's GFP signal to account for mitochondrial abundance) and GFP in microglia from the STR and MB of 3mo (STR N = 3 male, 3 female mice; MB 3 male, 3 female mice) and 18mo (STR N = 2 male, 3 female mice; MB 2 male, 4 female mice) mice. **(E-F)** Mitochondrial mass, number, and aspect ratio of NAc and VTA microglia during aging, normalized to mean values from 2-3mo animals (2-3mo N = 3 male, 4 female mice; 12-13mo N = 3 male, 1 female mice; 16-18mo N = 3 male, 2 female mice). **(G-H)** Microglial mitochondrial mass (mitochondrial volume / cell volume) and mitochondrial aspect ratio at 24hr after saline (N = 2 male, 3 female mice) or LPS injection (N = 3 male, 3 female mice). **(I-J)** Median Fluorescence Intensity of GFP and TMRM (normalized to individual cell's GFP signal to account for mitochondrial abundance) in STR and MB microglia from Saline (N = 3 male, 4 female mice), LPS 4hr (N= 3 male, 4 female mice), and LPS 24hr (N = 3 male, 4 female mice) mice. **(K-L)** FOV microglial volume across and FOV mitochondrial volume in CTR (N = 2 male, 2 female mice) and *MG-TFAMKO* (N = 3 male, 2 female mice) mice. Data was plotted as mean ± SEM for all graphs. Source data used to generate all graphs are provided as a Source Data file.

Supplementary Table 1: LPS Relative Gene Expression ANOVA Values.
Related to Figure 4B.

	Treatment		Region	
Gene	F Value	P Value	F Value	P Value
Tgfβ1	3.64818	0.03638	0.74976	0.39245
P2ry12	13.3998	0.0001	0.68065	0.41495
Csf1r	1.13966	0.34319	1.2454	0.27996
Cx3cr1	9.42003	0.000533	0.04288	0.83715
Cd68	4.11485	0.02483	5.06765	0.03076
Cd45	8.86009	0.00077	7.37401	0.01021
Itgam	14.09506	0.000246	1.07928	0.31341
Tnfα	10.23143	0.000317	0.01671	0.8979
Il1β	8.41188	0.00104	0.01627	0.89925
Micu1	5.40554	0.009	5.57555	0.02391
Cat	7.43629	0.00204	0.11711	0.73424
Ppargc1α	2.99706	0.06289	0.15601	0.69526
Nrf1	4.43215	0.01924	0.04594	0.83152
Mfn1	0.77771	0.46724	1.3191	0.25854
Dnm1l	4.8918	0.01338	0.31082	0.58072
Sod2	7.41671	0.00206	1.70341	0.20036
Tfam	2.1335	0.13357	1.72655	0.1974
Vdac1	12.4427	0.0001	0.06818	0.79554
Gpx1	20.79043	0.0001	1.01441	0.32076
Ndufa12	15.1893	0.0001	0.31724	0.57686
Atp5d	36.99749	0.0001	2.90216	0.09733
Cox4i1	12.11631	0.0001	1.69829	0.20102
Sdha	1.8012	0.18007	0.00647	0.93633

Supplementary Table 2: LPS Relative Gene Expression Post Hoc Analyses. Related to Figure 4B.

Genes		LPS 4hr vs Saline	LPS 24hr vs Saline	LPS 24hr vs LPS 4hr	MB vs STR
Tgfβ1	Probability	0.29856	0.03572	1	0.41278
	T value	-1.69206	-2.65327	-0.91161	-0.8289
P2ry12	Probability	0.00101	0.0001	1	0.45099
	T value	-3.97331	-4.8306	-0.76698	-0.76229
Csf1r	Probability	0.56513	1	1	0.33355
	T value	-1.37044	-0.94236	0.46004	-0.99552
Cx3cr1	Probability	0.00041798	0.45199	0.02272	0.95445
	T value	-4.27642	-1.46937	2.83451	-0.05753
Cd68	Probability	0.17688	1	0.03203	0.03952
	T value	-1.95212	0.75952	2.69744	-2.13869
Cd45	Probability	1	0.00219	0.00309	0.00733
	T value	0.05282	3.7024	3.58036	2.84717
Itgam	Probability	0.2025	0.000191597	0.01835	0.25439
	T value	1.95288	5.25978	3.12856	1.17964
Tnfα	Probability	0.000198161	0.117796	0.03916	0.9941
	T value	4.52848	1.9492	-2.61572	-0.00745
Il1β	Probability	0.00078693	0.42024	0.04286	0.98861
	T value	4.06006	1.50976	-2.57852	-0.01438
Micu1	Probability	0.27588	0.00708	0.4393	0.02479
	T value	-1.73266	-3.27919	-1.48524	-2.34559
Cat	Probability	0.00198	0.04203	0.71437	0.82245
	T value	-3.73851	-2.58666	1.2002	-0.22609
Ppargc1α	Probability	0.16869	0.09614	1	0.71262
	T value	-1.97473	-2.23293	-0.21646	-0.37134
Nrf1	Probability	0.21664	0.78056	0.01577	0.89431
	T value	1.85378	-1.24449	-2.97688	0.13382
Mfn1	Probability	1	0.76394	1	0.23403
	T value	-0.95289	-1.15815	-0.18361	1.21095
Dnm1l	Probability	0.02817	0.03151	1	0.58201
	T value	2.7491	2.70405	-0.0956	0.55561
Sod2	Probability	0.02418	0.00246	1	0.21081
	T value	2.80989	3.6624	0.78404	1.2747
Tfam	Probability	0.14411	0.35257	1	0.20397
	T value	2.04883	1.6048	-0.47402	-1.2945
Vdac1	Probability	0.09993	0.027	0.0001	0.64942
	T value	-2.2157	2.76603	4.93002	0.4585
Gpx1	Probability	0.06221	0.000865327	0.0001	0.2331
	T value	-2.42235	4.02733	6.3744	1.2134
Ndufa12	Probability	1	0.0001	0.00026155	0.66075
	T value	0.4567	4.98498	4.43509	-0.44264
Atp5d	Probability	1	0.0001	0.0001	0.13839
	T value	-0.26036	7.3228	7.44627	-1.51645
Cox4i1	Probability	1	0.000437904	0.00068819	0.25917
	T value	0.065	4.26059	4.11594	-1.14698
Sdha	Probability	0.32009	1	0.38157	0.98235
	T value	-1.65582	-0.0952	1.5624	0.02229

Supplementary Table 3: LPS Microglila- and Mitochondrial- Function Gene Correlation Values. Related to Figure 4D.

		Cd45			Il1β			Tnfα		
Genes		Saline	LPS 4hr	LPS 24hr	Saline	LPS 4hr	LPS 24hr	Saline	LPS 4hr	LPS 24hr
Atp5d	p-value	0.493624	0.137549	0.359852	0.257306	0.366931	0.13625	0.730913	0.775393	0.135199
	R value	0.334	0.524	0.233	0.131	0.322	0.501	0.218	0.098	0.493
Cox4i1	p-value	0.36897	0.06131	0.028433	0.405715	0.466862	0.206421	0.36897	0.648234	0.285042
	R value	0.44	0.612	0.669	0.395	0.274	0.401	0.443	-0.053	0.301
Ndufa12	p-value	0.409387	0.019198	0.004428	0.850599	0.074777	0.132485	0.80196	0.282537	0.062985
	R value	0.335	0.743	0.758	0.106	0.549	0.531	0.133	0.408	0.609
Sdha	p-value	0.510373	0.00215	0.008388	0.756231	0.141612	0.875422	0.950833	0.478784	0.86928
	R value	0.22	0.825	0.761	0.13	0.492	0.046	0.015	0.298	0.057
Nrf1	p-value	0.454638	0.007198	0.014085	0.994889	0.030809	0.830012	0.856943	0.03054	0.86928
	R value	0.323	0.787	0.717	0.055	0.668	-0.09	0.116	0.696	-0.062
Tfam	p-value	0.949681	0.016268	0.027111	0.826454	0.012704	0.522152	0.525826	0.071535	0.655121
	R value	0.047	0.712	0.69	-0.073	0.694	0.234	-0.197	0.636	0.152
Ppargc1α	p-value	0.637997	0.003019	0.205423	0.405715	0.59376	0.400037	0.405715	0.664901	0.522152
	R value	-0.189	0.679	0.429	-0.35	0.194	0.293	-0.336	0.169	0.208
Dnm1l	p-value	0.424293	0.06131	0.000262	0.912003	0.004815	0.206421	0.72725	0.004663	0.07025
	R value	0.326	0.587	0.891	-0.036	0.75	0.454	0.165	0.822	0.601
Mfn1	p-value	0.455014	1.92E-05	0.004428	0.637997	0.027085	0.652545	0.826454	0.211542	0.511684
	R value	0.3	0.83	0.799	-0.213	0.685	0.126	0.112	0.554	0.168
Micu1	p-value	0.752171	0.030809	0.285042	0.325813	0.179278	0.4977	0.856943	0.757082	0.285042
	R value	0.138	0.688	0.364	0.378	0.458	-0.347	0.063	0.144	-0.445
Vdac1	p-value	0.399239	0.030809	0.052616	0.493624	0.755757	0.253649	0.392043	0.400442	0.324445
	R value	0.422	0.691	0.59	0.087	0.105	0.381	0.363	-0.24	0.339
Sod2	p-value	0.295294	0.283494	0.830012	0.525465	0.00018	0.026746	0.295294	3.58E-06	0.003066
	R value	0.463	0.13	0.078	0.28	0.805	0.533	0.465	0.808	0.668
Cat	p-value	0.125641	0.155162	0.027111	0.295294	0.59376	0.561764	0.242937	0.146376	0.405422
	R value	0.621	0.5	0.402	0.443	-0.289	-0.315	0.491	-0.531	-0.409
Gpx1	p-value	0.171525	0.494331	0.001258	0.242937	0.308306	0.450881	0.431017	0.394667	0.298227
	R value	0.553	0.439	0.851	0.497	-0.408	0.294	0.304	-0.452	0.379

		Cx3cr1			P2ry12			Tgfβ1		
Genes		Saline	LPS 4hr	LPS 24hr	Saline	LPS 4hr	LPS 24hr	Saline	LPS 4hr	LPS 24hr
Atp5d	p-value	0.295294	0.603463	0.875422	0.206176	0.603463	0.228693	0.264582	0.065075	0.561764
	R value	0.239	0.193	0.024	0.207	0.191	-0.44	0.378	0.622	-0.229
Cox4i1	p-value	0.043146	0.091527	0.004428	0.249124	0.059409	0.612193	0.008057	0.044751	0.206421
	R value	0.736	0.515	0.762	0.541	0.537	0.169	0.849	0.653	0.425
Ndufa12	p-value	0.062521	0.645906	0.000728	0.049385	0.398998	0.522152	0.058573	0.00215	0.875422
	R value	0.673	0.144	0.489	0.688	0.261	-0.259	0.688	0.748	0.049
Sdha	p-value	0.050195	0.394667	0.001015	0.01295	0.227722	0.150088	0.053349	0.044665	0.001258
	R value	0.709	0.253	0.835	0.807	0.329	0.508	0.699	0.659	0.743
Nrf1	p-value	0.125641	0.725984	0.001015	0.053349	0.903248	0.137724	0.066148	0.073057	0.026746
	R value	0.59	-0.147	0.863	0.67	-0.068	0.54	0.65	0.606	0.675
Tfam	p-value	0.295294	0.648234	0.000112	0.035869	0.841112	0.206421	0.295294	0.072557	0.028563
	R value	0.4	-0.183	0.922	0.618	-0.087	0.453	0.421	0.64	0.683
Ppargc1α	p-value	0.455014	0.071535	0.001258	0.06157	0.001269	0.242876	0.525826	0.007817	0.135199
	R value	0.295	0.281	0.574	0.56	0.387	0.404	0.256	0.536	0.524
Dnm1l	p-value	0.249124	0.196433	0.094176	0.295294	0.319941	0.830012	0.295294	0.162525	0.612193
	R value	0.44	-0.489	0.565	0.425	-0.388	-0.126	0.391	0.46	0.127
Mfn1	p-value	0.404407	0.903248	0.001015	0.405715	0.755757	0.184554	0.409387	0.09787	0.027111
	R value	0.332	-0.082	0.864	0.32	-0.01	0.466	0.257	0.594	0.645
Micu1	p-value	0.008057	0.212226	0.007979	0.008839	0.117015	0.000783	0.052341	0.027085	0.000728
	R value	0.686	0.404	0.817	0.749	0.465	0.875	0.731	0.714	0.855
Vdac1	p-value	0.524534	0.000343	0.324445	0.36897	0.008035	0.851314	0.295294	0.12192	0.561764
	R value	0.067	0.61	0.418	0.009	0.607	0.055	0.231	0.544	0.195
Sod2	p-value	0.438239	0.09787	0.358364	0.535235	0.153205	0.094176	0.125641	0.00215	0.150088
	R value	0.344	-0.577	-0.415	0.239	-0.532	-0.58	0.538	0.352	-0.518
Cat	p-value	0.136261	3.46E-05	1.26E-05	0.264582	8.51E-05	0.000235	0.043146	0.196433	2.79E-08
	R value	0.59	0.936	0.87	0.477	0.927	0.905	0.733	0.486	0.926
Gpx1	p-value	0.038467	0.000343	0.027111	0.178605	0.00073	0.915929	0.058573	0.308306	0.358364
	R value	0.748	0.816	0.684	0.54	0.852	-0.002	0.683	0.399	0.315

Supplementary Table 4: LPS CD68 and Mitochondrial Function Gene Correlation Values. Related to Figure S4.

		Cd68		
Genes		Saline	LPS 4hr	LPS 24hr
Atp5d	p-value	0.242937	0.146376	0.568328
	R value	0.333	0.493	0.155
Cox4i1	p-value	0.136261	0.009325	0.021439
	R value	0.604	0.759	0.701
Ndufa12	p-value	0.171525	0.009708	0.027111
	R value	0.544	0.736	0.666
Sdha	p-value	0.018529	0.006375	0.015194
	R value	0.783	0.721	0.741
Nrf1	p-value	0.178605	0.240222	0.001499
	R value	0.549	0.408	0.844
Tfam	p-value	0.295294	0.155162	0.000728
	R value	0.413	0.48	0.883
Ppargc1α	p-value	0.326307	0.006855	0.000783
	R value	0.39	0.652	0.647
Dnm1l	p-value	0.295294	0.646276	0.097101
	R value	0.391	0.187	0.558
Mfn1	p-value	0.46778	0.141192	0.010282
	R value	0.231	0.425	0.758
Micu1	p-value	0.007659	0.009708	0.015224
	R value	0.873	0.737	0.641
Vdac1	p-value	0.317446	0.019198	0.568328
	R value	0.106	0.727	0.226
Sod2	p-value	0.438239	0.494331	0.358364
	R value	0.332	-0.291	-0.455
Cat	p-value	0.136644	0.008035	0.000103
	R value	0.575	0.772	0.678
Gpx1	p-value	0.125641	0.013089	4.58E-05
	R value	0.6	0.745	0.62
Il1β	p-value	0.848035	0.396429	0.928713
	R value	0.307	-0.096	0.14
Tnfα	p-value	0.888728	0.638932	0.844106
	R value	-0.041	-0.165	0.083
Tgfβ1	p-value	0.00144	0.033754	0.000626
	R value	0.858	0.635	0.666
Cd45	p-value	0.87144	0.112515	0.046827
	R value	0.069	0.479	0.635
Cx3cr1	p-value	4.05E-05	0.04008	2.76E-11
	R value	0.876	0.666	0.885
P2ry12	p-value	4.58E-05	0.00905	0.000348
	R value	0.879	0.777	0.398752

Supplementary Table 5: MG-TFAMKO FACS and relative expression ($2^{(-\Delta CT)}$) ANOVA Values. Related to Figure 8 and Figure S8.

	ANOVA - Genotype		ANOVA - Region		Posthoc Bonferroni - Genotype		Posthoc Bonferroni - Region		TFAMKO STR vs CTR STR		TFAMKO MB vs CTR MB	
Protein Level	F Value	P Value	F Value	P Value	T Value	Probability	T Value	Probability	T Value	Probability	T Value	Probability
CD45	0.02292	0.88118	**25.02099**	**0.0001**	-0.15139	0.88118	**-5.01669**	**0.0001**	-0.34282	1	0.12872	1
CX3CR1	1.68024	0.20965	**19.34603**	**0.000278**	1.29624	0.20965	**4.18028**	**0.000462**	2.0901	0.29753	-0.25694	1
FSC-A	**20.69067**	**0.0002**	1.15312	0.29568	**4.5487**	**0.0002**	1.01152	0.32385	**3.53081**	**0.01231**	2.89202	0.05408
SSC-A	**13.92572**	**0.00132**	0.39895	0.053479	**3.73172**	**0.00132**	0.52831	0.6031	**3.10839**	**0.03323**	2.16905	0.25384
Gene Level	F Value	P Value	F Value	P Value	T Value	Probability	T Value	Probability	T Value	Probability	T Value	Probability
Atp5d	**45.95018**	**0.0001**	2.01542	0.17111	**-6.77866**	**0.0001**	1.33835	0.1958	**-5.21762**	**0.00025**	**-4.36885**	**0.00178**
Cat	**9.26908**	**0.0064**	0.40487	0.5318	**-3.04452**	**0.0064**	-0.37529	0.71139	-1.02317	1	**-3.28242**	**0.02234**
Cd45	0.02413	0.8781	**15.02046**	**0.00095**	0.15535	0.8781	**-3.90608**	**0.00088**	0.21243	1	0.00727	1
Cd68	0.01473	0.9046	**24.29689**	**0.0001**	-0.12138	0.9046	**4.87956**	**0.0001**	-0.58592	1	0.41426	1
Cox4i1	**126.6341**	**0.0001**	0.07841	0.78235	**-11.25318**	**0.0001**	0.32718	0.74694	**-7.77651**	**0.0001**	**-8.13789**	**0.0001**
Csf1r	3.10295	0.09343	2.43891	0.13404	-1.76152	0.09343	1.63864	0.11692	-1.01638	1	-1.47479	0.93503
Cx3cr1	**17.01521**	**0.00053**	**12.58095**	**0.002**	**-4.12495**	**0.00053**	**3.36426**	**0.00309**	**-3.89155**	**0.00544**	-1.94201	0.39812
Dnm1l	**4.70405**	**0.04232**	0.8066	0.37981	**2.16888**	**0.04232**	-0.85845	0.40081	1.75281	0.5697	1.31445	1
Gpx1	4.03523	0.05825	21.4793	0.15831	-2.00879	0.0825	-1.3792	0.18306	-0.97209	1	-1.86877	0.45824
Ifnβ1	**16.45206**	**0.00062**	2.29609	0.14535	**4.05611**	**0.00062**	1.1731	0.25454	1.34674	1	**4.38947**	**0.0017**
Il1β	**12.24035**	**0.00226**	2.04	0.16864	**-3.49862**	**0.00226**	-1.16721	0.25685	-1.1297	1	**-3.65079**	**0.00954**
Itgam	0.66387	0.42479	0.27531	0.60556	-0.81478	0.42479	0.42126	0.67806	-1.04002	1	-0.11226	1
Mavs	**7.40066**	**0.01318**	0.00075	0.97845	**-2.72042**	**0.01318**	-0.05566	0.95616	-2.04043	0.32839	-1.80681	0.51517
Mb21d1 (cGas)	3.33367	0.08284	**6.31848**	**0.02062**	1.82584	0.08284	-2.22691	0.0376	2.63979	0.09427	-0.05767	1
Mfn1	0.14986	0.70276	0.99939	0.3294	0.38712	0.70276	-1.11031	0.28004	-0.12966	1	0.67713	1
Micu1	**38.60565**	**0.0001**	**6.75829**	**0.01714**	**-6.21334**	**0.0001**	**2.62282**	**0.0163**	**-4.45092**	**0.00147**	**-4.33608**	**0.00192**
Ndufa12	**7.17777**	**0.01442**	0.28616	0.59859	**-2.67914**	**0.01442**	0.63132	0.53498	-1.523	0.86049	-2.26587	0.20827
Nlrp3	0.04784	0.82908	0.61263	0.44297	-0.218873	0.82909	0.87232	0.39338	0.17378	1	-0.48312	1
Nrf1	**5.00741**	**0.03678**	0.8524	0.36688	**-2.23772**	**0.03678**	-0.79631	0.4352	-0.99648	1	-2.16815	0.25431
P2ry12	0.08108	0.77877	**9.97483**	**0.00494**	0.28747	0.77877	**3.09392**	**0.00572**	-0.25539	1	0.65808	1
Ppargc1α	**5.55677**	**0.02993**	0.075	0.78731	**2.35628**	**0.02993**	0.47328	0.6417	2.13737	0.27926	1.19633	1
Sdha	**8.99841**	**0.00708**	1.12328	0.30185	**-2.99973**	**0.00708**	1.18546	0.24973	-1.65856	0.67684	-2.58371	0.10644
Sod2	**4.7674**	**0.04109**	1.78966	0.19598	**-2.18344**	**0.04109**	-1.23455	0.23131	-1.03269	1	-2.05515	0.31896
Tfam probe 1	**128.37**	**0.0001**	1.31314	0.26535	**11.33005**	**0.0001**	1.39016	0.17976	**8.96526**	**0.0001**	**7.05784**	**0.0001**
Tfam probe 2	**87.30932**	**0.0001**	0.30509	0.58683	**9.34395**	**0.0001**	0.73623	0.47014	**7.34364**	**0.0001**	**5.87069**	**0.0001**
Tgfβ1	**15.69556**	**0.00077**	**9.22353**	**0.00651**	**-3.96176**	**0.00077**	**2.87826**	**0.00929**	**-3.64577**	**0.00965**	-1.95701	0.38671
Tlr9	2.81938	0.10869	1.89537	0.18381	-1.6791	0.10869	-1.36979	0.18594	-1.0766	1	-1.29801	1
Tmem173 (Sting)	0.68991	0.416	0.34889	0.56136	0.83061	0.416	-0.58405	0.56571	0.65007	1	0.52459	1
Tnfα	**29.54024**	**0.0001**	**7.40789**	**0.01314**	**-5.43509**	**0.0001**	**-2.53105**	**0.01987**	-2.88394	0.05506	**-4.80244**	**0.00065**
Vdac1	0.10673	0.7473	0.39367	0.53748	-0.32669	0.7473	-0.39797	0.69487	0.76612	1	-1.22813	1

Supplementary Table 6: Comparison of gene average fold changes ($2^{(-\Delta\Delta CT)}$) for MG-mitoGFP and MG-mitoGFP-TFAMKO samples following LPS challenge, relative to MG-mitoGFP saline treated samples. Related to Figure 4 and 8.

STRIATUM - Average Fold Changes (2^(-ΔΔCt)) to MG-mitoGFP + Saline

	MG-mitoGFP + Saline	MG-mitoGFP + 4hr LPS	MG-mitoGFP + 24hr LPS	MG-mitoGFP + Saline	MG-mitoGFP-TFAMWT + 12hr LPS	MG-mitoGFP-TFAMKO + 12hr LPS
Mitochondrial Related Genes	endo control: Gapdh	endo control: Gapdh	endo control: Gapdh	endo control: Gusb	endo control: Gusb	endo control: Gusb
Atp5d	1	1.0643	1.6086	1	0.7684	0.8749
Cat	1	0.7150	0.7701	1	0.3665	0.4199
Cox4i1	1	1.2138	1.4266	1	0.6196	0.8524
Dnm1l	1	1.6079	1.3928	1	1.0166	1.0736
Gpx1	1	0.7881	1.5620	1	0.9054	0.9643
Mavs				1	0.5385	0.5734
Mfn1	1	1.0667	0.9384	1	0.6366	0.7581
Micu1	1	0.8633	0.6883	1	0.3600	0.4794
Ndufa12	1	1.2943	2.0937	1	1.1686	1.2309
Nrf1	1	1.3074	0.9263	1	0.5448	0.6396
Ppargc1α	1	0.8476	0.6484			
Sdha	1	0.8998	0.9342	1	0.6261	0.7414
Sod2	1	2.3658	2.2959	1	1.6468	2.1885
Tfam probe 1				1	0.7573	0.4564
Tfam probe 2	1	1.4357	1.2646	1	0.5454	0.3448
Vdac1	1	0.9288	1.2152	1	0.6042	0.7660
Microglial Related Genes						
Cd45	1	1.1367	1.3506	1	0.5219	0.5597
Cd68	1	0.8428	1.0707	1	0.3137	0.4081
Csf1r	1	0.9567	0.9133	1	0.4170	0.4551
Cx3cr1	1	0.5828	0.8266	1	0.2943	0.2885
Ifnβ1				1	1.1322	0.8297
Il1β	1	79.2113	37.1183	1	17.9284	16.9704
Itgam	1	1.4364	1.5346	1	0.6232	0.7448
Mb21d1 (cGas)				1	1.1895	1.2916
Nlrp3				1	0.8822	1.0850
P2ry12	1	0.4974	0.3403	1	0.1037	0.1020
Tgfβ1	1	0.8933	0.7280	1	0.3126	0.3276
Tlr9				1	0.5578	0.6234
Tmem173 (Sting)				1	0.3207	0.3293
Tnfα	1	100.1634	37.9237	1	21.0308	35.9473

MIDBRAIN - Average Fold Changes (2^(-ΔΔCt)) to MG-mitoGFP + Saline

	MG-mitoGFP + Saline	MG-mitoGFP + 4hr LPS	MG-mitoGFP + 24hr LPS	MG-mitoGFP + Saline	MG-mitoGFP-TFAMWT + 12hr LPS	MG-mitoGFP-TFAMKO + 12hr LPS
Mitochondrial Related Genes	endo control: Gapdh	endo control: Gapdh	endo control: Gapdh	endo control: Gusb	endo control: Gusb	endo control: Gusb
Atp5d	1	0.9707	1.5187	1	0.8185	0.8055
Cat	1	0.6389	0.7711	1	0.4019	0.4204
Cox4i1	1	0.9812	1.4290	1	0.6812	0.7862
Dnm1l	1	1.5099	1.5534	1	1.0222	1.1646
Gpx1	1	0.6631	1.6610	1	0.7774	0.8051
Mavs				1	0.5048	0.6817
Mfn1	1	0.9863	0.9047	1	0.5513	0.6506
Micu1	1	0.9840	0.8309	1	0.5354	0.5724
Ndufa12	1	1.1208	2.0564	1	1.2315	1.2866
Nrf1	1	1.3079	0.8748	1	0.5526	0.5607
Ppargc1α	1	1.2643	0.9411			
Sdha	1	0.9215	1.0947	1	0.7271	0.7724
Sod2	1	2.9739	3.3247	1	3.4827	3.5568
Tfam probe 1				1	0.7846	0.4604
Tfam probe 2	1	1.2224	1.1549	1	0.5519	0.3341
Vdac1	1	0.8132	1.2727	1	0.6335	0.7101
Microglial Related Genes						
Cd45	1	0.9856	1.3504	1	0.4658	0.4768
Cd68	1	0.7738	1.1420	1	0.3439	0.3625
Csf1r	1	0.8517	0.8822	1	0.3997	0.4461
Cx3cr1	1	0.5303	0.8872	1	0.3374	0.3277
Ifnβ1				1	0.5462	0.4864
Il1β	1	196.3872	39.4252	1	61.5999	31.5696
Itgam	1	1.4656	1.8833	1	0.8542	0.9547
Mb21d1 (cGas)				1	1.2161	1.3084
Nlrp3				1	1.3447	1.3352
P2ry12	1	0.4481	0.3843	1	0.0900	0.1108
Tgfβ1	1	0.8748	0.7794	1	0.3271	0.3190
Tlr9				1	0.3916	0.4157
Tmem173 (Sting)				1	0.3253	0.3055
Tnfα	1	63.6890	25.8358	1	26.2134	26.6842

Supplementary Table 7: MG-mitoGFP-TFAMKO 12hr LPS FACS and fold change ($2^{(-\Delta\Delta CT)}$) ANOVA Values. Related to Figure 8 and Figure S8.

	ANOVA - Genotype		ANOVA - Region		Posthoc Bonferroni - Genotype		Posthoc Bonferroni - Region		TFAMKO STR vs CTR STR		TFAMKO MB vs CTR MB	
Protein Level	F Value	P Value	F Value	P Value	T Value	Probability	T Value	Probability	T Value	Probability	T Value	Probability
CD45	0.35092	0.56187	**28.09286**	**0.0001**	0.59239	0.56187	**-5.47722**	**0.0001**	0.18451	1	0.65326	1
CX3CR1	1.0023	0.33166	**17.12375**	**0.00077**	1.00115	0.33166	**4.31983**	**0.00053**	1.04191	1	0.37393	1
GFP	0.68349	0.42055	3.70828	0.0721	-0.82673	0.42055	-2.0286	0.05948	-0.80352	1	-0.36565	1
TMRM (norm to GFP)	0.000133	0.99091	2.99229	0.1029	0.01157	0.99091	1.74114	0.10085	-0.07616	1	0.09253	1
FSC-A	**15.99502**	**0.00103**	0.00217	0.96338	**-3.9938**	**0.00103**	-0.10335	0.91897	**-3.02113**	**0.04868**	-2.63485	0.1081
SSC-A	3.03955	0.10044	0.43762	0.51769	1.74343	0.10044	0.65455	0.52206	1.16136	1	1.30422	1
Gene Level	F Value	P Value	F Value	P Value	T Value	Probability	T Value	Probability	T Value	Probability	T Value	Probability
Atp5d	0.72773	0.40798	0.031	0.86276	-0.85307	0.40798	0.05531	0.95667	-1.37387	1	0.16745	1
Cat	1.36818	0.26165	0.34219	0.56788	-1.16969	0.26165	-0.65222	0.52483	-1.22933	1	0.42486	1
Cd45	0.30415	0.58999	2.47447	0.13803	-0.5515	0.58999	1.54885	0.14372	-0.60495	1	-0.17498	1
Cd68	**4.75047**	**0.04686**	0.08683	0.77257	**-2.17956**	**0.04686**	0.13311	0.896	-2.57458	0.13224	-0.50778	1
Cox4i1	**9.4789**	**0.00817**	0.00174	0.96731	**-3.07878**	**0.00817**	-0.08819	0.93098	-3.00025	0.05728	-1.3538	1
Csf1r	2.10639	0.16872	0.20527	0.65744	-1.45134	0.16872	0.47173	0.64439	-0.9261	1	-1.12641	1
Cx3cr1	0.02797	0.86958	0.78802	0.3897	0.16723	0.86958	-0.89802	0.38436	0.08798	1	0.14852	1
Dnm1l	1.99438	0.17972	0.46871	0.50476	-1.41223	0.17972	-0.62125	0.54442	-0.57078	1	-1.4264	1
Gpx1	0.19037	0.66926	2.09591	0.16972	-0.43632	0.66926	1.43919	0.17208	-0.41957	1	-0.19748	1
Ifnβ1	1.38512	0.25885	**9.10928**	**0.00921**	1.17691	0.25885	**3.12508**	**0.00745**	1.38953	1	0.27487	1
Il1β	1.18581	0.29457	4.19297	0.05983	1.08895	0.29457	-2.17465	0.04729	0.04761	1	1.4924	0.9467
Itgam	**9.71587**	**0.00757**	**38.28983**	**0.0001**	**-3.11703**	**0.00757**	**-6.25963**	**0.0001**	-2.41403	0.1803	-1.99412	0.39595
Mavs	**7.42879**	**0.01641**	0.92349	0.35287	**-2.72558**	**0.01641**	-0.76275	0.45827	-0.63566	1	**-3.21889**	**0.0371**
Mb21d1 (cGas)	0.71785	0.4111	0.03603	0.85217	-0.84726	0.4111	-0.19579	0.84759	-0.62932	1	0.56888	1
Mfn1	**5.01149**	**0.04194**	3.81642	0.07103	**-2.23864**	**0.04194**	1.94061	0.07272	-1.74185	0.62071	-1.42406	1
Micu1	**4.60747**	**0.04984**	**13.55873**	**0.00246**	**-2.1465**	**0.04984**	**-3.83155**	**0.00183**	-2.31719	0.21691	-1.71174	0.65398
Ndufa12	0.48029	0.49963	0.48985	0.49546	-0.69303	0.49963	-0.70896	0.48999	-0.51978	1	-0.46031	1
Nlrp3	0.29591	0.59502	4.02278	0.06461	-0.54397	0.59502	-2.0849	0.05586	-0.80716	1	0.03787	1
Nrf1	0.44038	0.51772	0.21063	0.65332	-0.66361	0.51772	0.39932	0.69568	-0.86441	1	-0.07408	1
P2ry12	0.63472	0.43893	0.04136	0.84178	-0.7967	0.43893	0.30901	0.76186	0.09682	1	-1.22352	1
Sdha	2.89461	0.11097	1.95487	0.18382	-1.70136	0.11097	-1.48974	0.15874	-1.72712	0.63679	-0.67896	1
Sod2	0.14255	0.71142	3.85925	0.06964	-0.37756	0.71142	-2.00879	0.06425	-0.46973	1	-0.06423	1
Tfam probe 1	**35.54773**	**0.0001**	0.08884	0.77004	**5.96219**	**0.0001**	-0.32467	0.75023	**4.05931**	**0.00703**	**4.37251**	**0.00383**
Tfam probe 2	**22.46606**	**0.00032**	0.00237	0.96186	**4.73984**	**0.00032**	0.0272	0.97868	**3.21378**	**0.03748**	**3.48936**	**0.02166**
Tgfβ1	0.0166	0.89933	0.01159	0.91578	-0.12883	0.89933	-0.15594	0.87831	-0.4003	1	0.20992	1
Tlr9	1.05088	0.32269	**18.2921**	**0.00077**	-1.02513	0.32269	**4.25042**	**0.00081**	-1.06107	1	-0.38868	1
Tmem173 (Sting)	0.01694	0.89829	0.0505	0.82544	0.13017	0.89829	0.18902	0.85279	-0.14263	1	0.32671	1
Tnfα	1.83805	0.19665	0.12926	0.72457	-1.35575	0.19665	0.21946	0.82946	-1.85866	0.50535	-0.05866	1
Vdac1	**7.44427**	**0.01632**	0.09222	0.76584	**-2.72842**	**0.01632**	0.19653	0.84702	-2.6189	0.12132	-1.23967	1

Supplementary Table 8: Primers and probes used for qPCR experiments. Related to Figures 4, S4, 8, and S8.

Gene	TaqMan Assay ID or custom probe	Forward Primer	Reverse Primer
Aldh1l1	Mm03048957_m1		
Atp5d	Mm00502864_m1		
Cat	Mm00437992_m1		
Cd45 (Ptprc)	Mm01293577_m1		
Cd68	Mm03047343_m1		
Cox4i1	Mm01250094_m1		
Csrf1	Mm01266652_m1		
Cx3cr1	Mm00438354_m1		
Dnm1l	Mm01342903_m1		
Gapdh	MGB - CTCATGACCACAGTCCA - VIC	GACAACTTTGGCATTGTGGAA	CACAGTCTTCTGGGTGGCAGTGA
Gpx1	Mm04207457_g1		
Ifnβ1	Mm00439552_s1		
Il1β	Mm00434228_m1		
Itgam	Mm00434455_m1		
Mavs	Mm00523170_m1		
Mb21d1 (cGas)	Mm01147496_m1		
Mfn1	Mm00612599_m1		
Micu1	Mm00522783_m1		
Nrf1	Mm01135606_m1		
Ndufa12	Mm01240336_m1		
Nlrp3	Mm00840904_m1		
Olig1	Mm00497537_s1		
Olig2	Mm01210556_m1		
P2ry12	Mm00446026_m1		
Ppargc1α	Mm01208835_m1		
Ppp1r1b	Mm00454892_m1		
Sdha	Mm01352366_m1		
Slc1a2	Mm01275814_m1		
Slc32a1	Mm00494138_m1		
Sod2	Mm01313000_m1		
Tfam probe 1	Mm00627275_g1		
Tfam probe 2	Mm01148667_m1		
Tgfβ1	Mm01178820_m1		
Tlr9	Mm07299609_m1		
Tmem173 (Sting)	Mm01158117_m1		
Tnfα	Mm00443258_m1		
Vdac1	Mm00834272_m1		
