# Supplementary material for: Dynamic changes in mitochondria support phenotypic flexibility of microglia
Source: Nat Commun. 2025 Dec 12;16:11103. doi: 10.1038/s41467-025-66709-5 (PMC12700904; doi:10.1038/s41467-025-66709-5)
Supplement: Supplementary file 2 — Reporting Summary [file 41467_2025_66709_MOESM2_ESM.pdf]

nature portfolio

Corresponding author(s): Lindsay De Biase,
Last updated by author(s): 9/22/2025

Reporting Summary

Nature Portfolio wishes to improve the reproducibility of the work that we publish. This form provides structure for consistency and transparency in reporting. For further information on Nature Portfolio policies, see our Editorial Policies and the Editorial Policy Checklist.

Statistics

For all statistical analyses, confirm that the following items are present in the figure legend, table legend, main text, or Methods section.

n/a	Confirmed	
☐	☒	The exact sample size (*n*) for each experimental group/condition, given as a discrete number and unit of measurement
☐	☒	A statement on whether measurements were taken from distinct samples or whether the same sample was measured repeatedly
☐	☒	The statistical test(s) used AND whether they are one- or two-sided<br>*Only common tests should be described solely by name; describe more complex techniques in the Methods section.*
☒	☐	A description of all covariates tested
☐	☒	A description of any assumptions or corrections, such as tests of normality and adjustment for multiple comparisons
☐	☒	A full description of the statistical parameters including central tendency (e.g. means) or other basic estimates (e.g. regression coefficient) AND variation (e.g. standard deviation) or associated estimates of uncertainty (e.g. confidence intervals)
☐	☒	For null hypothesis testing, the test statistic (e.g. *F*, *t*, *r*) with confidence intervals, effect sizes, degrees of freedom and *P* value noted<br>*Give P values as exact values whenever suitable.*
☒	☐	For Bayesian analysis, information on the choice of priors and Markov chain Monte Carlo settings
☒	☐	For hierarchical and complex designs, identification of the appropriate level for tests and full reporting of outcomes
☒	☐	Estimates of effect sizes (e.g. Cohen's *d*, Pearson's *r*), indicating how they were calculated

Our web collection on statistics for biologists contains articles on many of the points above.

Software and code

Policy information about availability of computer code

Data collection	qPCR data was collected using QuantStudio5. FACS FCS files were analyzed in FlowJo v10.8 to obtain fluorescence measures.
Data analysis	Imaris (Bit plane) software v9.5 and v10.0 for analysis of confocal images. 3Dmorph, a previously published automated analysis pipeline by York et al., was adapted to analyze microglial motility. This modified code is available on CodeOcean. To gain an understanding of the cell complexity, we used the Simple Neurite Tracer plugin in image j by Arshadi et al. To analyze mitochondrial motility, the isolated MitoEGFP channel movies were analyzed in Imaris (Bit Plane) software v10.0. FlowJo v10.8 was used to analyze FACS data. OriginPro v and R version 4.3.3 were used for data analysis and statistics.

For manuscripts utilizing custom algorithms or software that are central to the research but not yet described in published literature, software must be made available to editors and reviewers. We strongly encourage code deposition in a community repository (e.g. GitHub). See the Nature Portfolio guidelines for submitting code & software for further information.

Data

Policy information about availability of data

All manuscripts must include a data availability statement. This statement should provide the following information, where applicable:

- Accession codes, unique identifiers, or web links for publicly available datasets
- A description of any restrictions on data availability
- For clinical datasets or third party data, please ensure that the statement adheres to our policy

Source data are provided with this paper as a source data file. Source data used to prepare all figures in this study have been deposited in FigShare database. Raw

data files are available to researchers wishing to carry out additional analyses or validation analyses for non-commercial purposes. Access to raw data files can be obtained by contacting Dr. Lindsay De Biase, ldebiase@mednet.ucla.edu.

Research involving human participants, their data, or biological material

Policy information about studies with human participants or human data. See also policy information about sex, gender (identity/presentation), and sexual orientation and race, ethnicity and racism.

Reporting on sex and gender	N/A
Reporting on race, ethnicity, or other socially relevant groupings	N/A
Population characteristics	N/A
Recruitment	N/A
Ethics oversight	N/A

Note that full information on the approval of the study protocol must also be provided in the manuscript.

Field-specific reporting

Please select the one below that is the best fit for your research. If you are not sure, read the appropriate sections before making your selection.

[x] Life sciences [ ] Behavioural & social sciences [ ] Ecological, evolutionary & environmental sciences

For a reference copy of the document with all sections, see nature.com/documents/nr-reporting-summary-flat.pdf

Life sciences study design

All studies must disclose on these points even when the disclosure is negative.

Sample size	Selection of sample size was informed by power analyses based on existing published data to generate group sizes that ensure statistical power (0.9) is sufficient for detecting statistically significant differences between groups (P < 0.05).
Data exclusions	No data points were excluded from analysis.
Replication	In some cases, we analyzed microglial properties that we previously reported as showing regional differences (e.g. cell density and tissue coverage) to confirm that they are still evident in these experimental cohorts of mice. Results from distinct qPCR experiments within the study were compared with one another to determine consistency. qPCR experiments were run with two endogenous housekeeping genes. qPCR was used to validate cell purity of purified microglia as done in previous studies. Success of TFAM knockdown was verified in each cohort of TFAM-KO mice.
Randomization	Mice were randomly assigned to saline, LPS 4hr, or LPS 24hr groups. Sort order for samples analyzed by flow cytometry was systematically varied so as to avoid introducing bias from processing time.
Blinding	Investigators were blinded during histology analysis of saline versus LPS treated mice. Investigators were blinded during histology analysis of TFAM-KO vs control mice. Investigators were not blinded to sample ID during flow cytometry and qPCR based analyses as analysis thresholds are set by FMO controls and Ct values of target and endogenous control genes and do not require manual thresholding as is the case for histological analyses.

Reporting for specific materials, systems and methods

We require information from authors about some types of materials, experimental systems and methods used in many studies. Here, indicate whether each material, system or method listed is relevant to your study. If you are not sure if a list item applies to your research, read the appropriate section before selecting a response.

Materials & experimental systems

n/a	Involved in the study	
☐	☒	Antibodies
☒	☐	Eukaryotic cell lines
☒	☐	Palaeontology and archaeology
☐	☒	Animals and other organisms
☒	☐	Clinical data
☒	☐	Dual use research of concern
☒	☐	Plants

Methods

n/a	Involved in the study	
☒	☐	ChIP-seq
☐	☒	Flow cytometry
☒	☐	MRI-based neuroimaging

Antibodies

Antibodies used	Primary antibodies used include rabbit anti- Iba1 (1:800; Wako, catalog #019-19741), rat anti-CD68 (1:200; clone FA11, AbD Serotec, catalog #MCA1957), chicken anti-TH (1:500; Aves, catalog #TYH). Secondary antibody combinations include rabbit AlexaFluor-647, chicken AlexaFluor-594, rat AlexaFluor-594, or chicken AlexaFluor-405 (used at 1:1000; all raised in donkey; Jackson ImmunoResearch Laboratories). Flow Cytometry antibodies included anti-CD45 (1:400; Biolegend, 147703), anti-P2RY12 (1:500; Biolegend, 848005), and anti-CX3CR1 (1:800; Biolegend, 149023).
Validation	Antibodies for histology were validated by including staining controls with no primary antibody. Antibodies for flow cytometry were verified by including fluorescence minus one (FMO) controls. In the case of antibodies for gating microglia, accurate targeting of the cell population was also verified by testing on mice in which microglia express genetically encoded fluorophore (e.g. CX3CR1-EGFP mice).

Animals and other research organisms

Policy information about studies involving animals; ARRIVE guidelines recommended for reporting animal research, and Sex and Gender in Research

Laboratory animals	This study used mice on the C57Bl6 background between 1.5-18 months of age
Wild animals	None used.
Reporting on sex	Both male and female mice were used in this study with balanced representation of each sex to the extent possible.
Field-collected samples	None used.
Ethics oversight	All procedures followed animal care guidelines approved by the University of California, Los Angeles Chancellor's Animal Research Committee. (UCLA Animal Research Committee (ARC) and UCLA institutional animal care and use committee (IACUC)).

Note that full information on the approval of the study protocol must also be provided in the manuscript.

Plants

Seed stocks	N/A
Novel plant genotypes	N/A
Authentication	N/A

Flow Cytometry

Plots

Confirm that:

- [x] The axis labels state the marker and fluorochrome used (e.g. CD4-FITC).
- [x] The axis scales are clearly visible. Include numbers along axes only for bottom left plot of group (a 'group' is an analysis of identical markers).
- [x] All plots are contour plots with outliers or pseudocolor plots.
- [x] A numerical value for number of cells or percentage (with statistics) is provided.

Methodology

Sample preparation	Mice were deeply anesthetized with isoflurane and transcardially perfused with 10 ml of chilled 1M PBS. The brain was rapidly removed, cut coronally with a razor blade using anatomical landmarks and midbrain and striatum were dissected free. Samples were thoroughly minced with razor blade on a chilled glass surface and transferred to 1.7mL tubes containing 1 mL Hibernate A Low-Fluorescence (Transnetyx Tissue, Half100). Samples were then mechanically dissociated using gentle sequential trituration with fire-polished glass pipettes of decreasing diameter. Cell suspensions were pelleted via centrifugation and resuspended in 1M PBS + Debris Removal Solution (Miltenyi, 130-109-398). Debris removal was carried out according to manufacturer's instructions. Following debris removal, samples were resuspended in small volumes of 1M PBS and incubated with primary antibodies on ice for 20min. Tetramethylrhodamine (TMRM) (Biotium, 70017) was used in some experiments to assess mitochondrial membrane potential. For these experiments, samples incubated with TMRM 50nM for 20 min at room temperature, prior to the antibodies step. Following incubation, samples were washed with 1xPBS and filtered through a 35mm filter (Falcon, 352235) prior to sorting. Throughout the experiment, samples were kept at 4°C on ice.
Instrument	Samples were sorted and data was collected using FACS Aria I cell sorters (BD Biosciences)
Software	FACS FCS files were analyzed in FlowJo v10.8.
Cell population abundance	Between 2,000-10,000 cells in the target cell population were gated for analysis and collection
Gating strategy	The population of cells containing microglia could be readily identified based on forward scattering (FSC) and side scattering (SSC) properties. A gating strategy based on FSC and SSC width and height was used to select single cells and reduce dead cells and doublets. Microglial cells within this population were then identified and sorted based on combinations of microglial antibodies (CD45, P2RY12, and CX3CR1). Placement of gates was verified using unstained samples as well as single color controls and fluorescence minus one (FMO) controls.

- [x] Tick this box to confirm that a figure exemplifying the gating strategy is provided in the Supplementary Information.
